# What Happens at Work Comes Home

**DOI:** 10.3390/healthcare8030350

**Published:** 2020-09-21

**Authors:** Anna Stowe Alrutz, Stephen Buetow, Linda D. Cameron, Peter Kenneth Huggard

**Affiliations:** 1 School of Population Health, Faculty of Medical and Health Sciences, University of Auckland, Auckland 1142, New Zealand; s.buetow@auckland.ac.nz (S.B.); p.huggard@auckland.ac.nz (P.K.H.); 2 Department of Psychology, Faculty of Science, University of Auckland, Auckland 1142, New Zealand; l.cameron@auckland.ac.nz; 3 Department of Psychological Sciences, School of Social Sciences, Humanities and Art, University of California, Merced, CA 95343, USA; 4 Institute of Rural Health, Division of Health Sciences Idaho State University, Pocatello, ID 83209, USA

**Keywords:** ambulance, defence force, emergency responder, fire service, first responder, military, New Zealand, partner, police, secondary trauma

## Abstract

Emergency responders (police, fire, ambulance and defence force personnel) risk exposure to dangerous and traumatic events, and the possible subsequent development of post-traumatic stress disorder. Consequently, partners of these emergency responders risk developing secondary traumatic stress (STS) from vicarious exposure to the trauma through communication and engagement with their responders. A mixed-methods study of the partners of emergency responders in New Zealand examined the extent of such partner-associated STS. This article focuses on two research questions: to what extent were risk factors for STS identified within that population, and what did the participants believe may help them to mitigate the impact of STS. An online anonymous survey was developed and eligible participants completed a 17-item STS scale, a social support measure, and answered several open-ended questions. Of the 646 participants, twenty percent appear to be experiencing intrusion, arousal, and avoidance symptoms related to the trauma experienced by their responder. Almost half stated they have little or no emotional/informational support related to their responder’s work. Thematic analysis of free-text responses identified the need for additional support and more direct communication/engagement from the organisations for partners to navigate their experiences of STS and the level of social support received and required. The authors conclude with recommendations to emergency responder organisations.

## 1. Introduction

### 1.1. Literature Review

In 2014/2015, New Zealand had over 33,000 defence and emergency responders; this included about 11,000 regular and reserve personnel in the New Zealand Defence Force (NZDF); around 9000 police; almost 9000 volunteer and paid firefighters; and around 4000 volunteer and paid ambulance officers. Their job of helping to protect the public exposes them to many life-threatening and dangerous situations and puts them at risk of experiencing traumatic stress [1,2,3,4]. A study looking at the psychological health of New Zealand emergency services organisations, including firefighters, police and ambulance services, found that “stressful job characteristics and psychological outcomes are not necessarily unique to any one service” [1] (p. 238).

While defence and emergency response organisations pay increasing attention to mitigating the potential of these roles to cause employee traumatic stress, limited attention has been paid to the impact on the employee’s partners and spouses [5,6,7]. These partners risk developing secondary traumatic stress (STS) from engaging with their responder’s stress reactions [5,8,9]. In New Zealand it is estimated that about half of the defence and emergency responders have partners, which means approximately 16,000 partners associated with these organisations are at risk of becoming traumatised. Studies of secondary trauma in other groups (e.g., social workers, counsellors, and psychologists) indicate that such secondary trauma is both preventable and treatable [10,11,12].

Social support has long been identified as a method of reducing the negative impact of stress on health as well as decreasing psychological risks, morbidity, and mortality [13,14,15]. Support from significant others, such as partners and spouses, can increase the likelihood of individuals seeking mental health care [16,17]. In reporting a positive outcome of such support, Hoyt and colleagues [18] found that military members who disclosed traumatic events to spouse, friends, and family exhibited fewer primary traumatic stress symptoms than those who disclosed to therapists. The authors suggested providing family groups with education programmes to become more helpful listeners. Thus, coordinated social support from the organisation, peers, and partners plays an important role in how responders manage their own trauma [19,20,21,22].

### 1.2. Brief Timeline

The effects of primary trauma on partners of military service members have been discussed since the early 1980s in publications such as Stress and the Family [23] and ‘Detoxification’ of Vietnam War trauma, a combined family individual approach [24]. During that time, awareness has grown that both family-focused social support and organisational support are key parts of the solution but progress has been slow. Figley reported in 1985 that “the family in particular and the entire social support system in general serve as an antidote to post-traumatic stress disorder” [25] (p. 284). Yet twenty years later, he was asking the organisations who the spouses could turn to for help [6] and again in 2016, he was still pointing out organisational dysfunctions that impede partners from getting help, stating that the “current organizational and leadership structure as it pertains to mental healthcare is critically failing military personnel and their families” [26] (p. 71).

### 1.3. Purpose of the Study

To improve understanding of the lives of the partners of New Zealand’s defence and emergency responders, the present study was designed to investigate how these partners respond to high-risk, work-induced stress experienced by their responder. Findings are presented for two research questions: To what extent were risk factors for STS identified within that population? What did the participants believe may help them to mitigate the impact of such stress? Two quantitative measures were used to address the first research question while qualitative analysis of two open-ended questions was used to address the second research question.

## 2. Methods

### 2.1. Recruitment and Participation

A mixed methods study was conducted across all regions of New Zealand in accordance with the protocols of the University of Auckland Human Participants Ethics Committee on five December, 2013 (reference number 010832). The study delivered a cross-sectional survey via an online, self-administered, anonymous, questionnaire, on a survey research platform called LimeSurvey version 2.0 [27]. Eligible participants were the self-defined partners, spouses, girlfriends/boyfriends of current New Zealand Defence, Police, Fire and Ambulance personnel. Recruitment was through official organisational channels such as newsletters, support group representatives and online portals as well as grassroots programs set up by/for partners such as Facebook, blogs, and groups associated with the defence/emergency responder organisations. In total, 945 participants self-reported meeting eligibility criteria for the survey and gave their informed consent to participate in the survey. Participants were excluded from analyses if they did not complete the entire survey (251 individuals); they indicated that their defence/emergency responder partner had not experienced at least one stressful event from the Life Events Checklist (LEC-5) [28] screening for traumatic events (26 individuals); and they did not complete the partner adaptation to the secondary traumatic stress scale (four individuals). These exclusions resulted in a final convenience sample of 664 partners.

### 2.2. Participant Demographics

Participants were from each of the 20 District Health Board regions in New Zealand, 583 identified as females, 74 as males, and seven individuals chose not to answer the question. They were aged 18 to 73 years (mean = 39.8 years) and had been together as a couple with their emergency responder partner from several months to 54 years (mean = 14.2 years). Their defence/emergency responder had been working in their jobs for a mean of 14.6 years, with four percent currently working for more than one organisation. The proportions of partners from each organisation represented in this study mirrored very closely those of the respective organisations. Of the 664 partners who participated in this study, 27 percent were partners of police officers, 33 percent were partners of firefighters, 31 percent were NZDF members’ partners and 13 percent were ambulance service partners.

### 2.3. Adaptation to the Secondary Traumatic Stress Scale (Adapted STS Scale)

The 17-item Secondary Traumatic Stress Scale (STSS) [29], was modified for the current study. Guided by the DSM-IV, the STSS was designed to identify the frequency of intrusion, avoidance, and arousal symptoms experienced because of indirect exposure to traumatic events in social workers. Response options were based on a five-point Likert Scale ranging from ‘never’ to ‘very often’.

The STSS is an internally reliable measure of STS, Cronbach’s α = 0.93 [29] and Cronbach’s α = 0.94 [30]. Although the scale is not used as a PTSD diagnostic tool, it indicates the proportion of participants meeting the symptomatic criteria for possible PTSD and can be examined using a cut-off score. The STSS was validated in social work populations [29,30] and has been widely used in various cultures [31,32] and participant groups including nurses [33], counsellors [34], midwives [35], healthcare providers [36], law enforcement investigators [37], and domestic violence advocates [38]. This scale was developed to evaluate STS with workers, minor modifications to the scale questions and directions made it fit to use with partners of emergency responders. The adapted scale was reviewed and approved by the main author of the STSS. Eight questions were modified to describe secondary symptoms in partners of responders. For example, ‘I had disturbing dreams about my work with clients’ became ‘I had disturbing dreams about my emergency responder’s stressful experience(s)’.

### 2.4. Emotional/Informational Support Measure

To evaluate informational and emotional support available to the partners of responders, a portion of the widely used Medical Outcomes Study (MOS) Social Support Survey [39] was adapted for use in this study. The MOS comprises four separate social support subscales: emotional/informational support, tangible support, affectionate support, and positive social interaction. These can be evaluated individually or as a group. The subscale for information and emotional support showed high convergent and discriminant validity with a Cronbach’s α = 0.96 and that portion was chosen for this study. The current study modified the wording for the directions and the eight chosen items on the scale so that they were appropriate for the study population. For example: “someone who understands your problems” became “someone who understands your problems with your emergency responder’s work”. Items for the MOS subscale can be found in Section 3.2.

### 2.5. Thematic Analysis

Analysis of the online survey’s qualitative responses was guided by Attride-Stirling’s [40] thematic networks. This framework was used for condensing/dissecting the raw text data into thematic categories. NVIVO 10 (QSR International, Melbourne, Australia) a qualitative data management software programme, was used to store, classify, sort, and examine relationships in qualitative responses. The online anonymous responses to two open-ended questions were downloaded directly into the programme and common or related codes (labelling text relevant to answering the study questions) were grouped into themes.

## 3. Results

### 3.1. Adapted Secondary Traumatic Stress Scale

The Adapted STS Scale consisted of 17 items and, in this study, yielded Cronbach’s α = 0.94. Results for the Adapted STS Scale were analysed using cut-off scores recommended for the original STS Scale [41]. The table below presents cut-off scores which place individuals into one of five categories based on their responses. The scores range from 17 to 74. Individuals achieving a cumulative score of less than 28 are interpreted as having ‘little or no risk of secondary trauma’; scores between 28 and 37 indicate ‘mild risk of secondary trauma’; scores between 38 and 43 indicate ‘moderate risk of secondary trauma’; with ‘high risk of secondary trauma’ indicated if the scores were between 44 and 48. Finally, any scores of 49 or above indicate ‘severe risk of secondary trauma’. Table 1 presents scores for the individual categories and a combined score for the Moderate/High/Severe risk categories indicating possible secondary trauma.

This table shows that 20 percent of participants appeared to struggle with intrusive, arousal, and avoidance thoughts about the trauma experienced by their emergency responder.

### 3.2. Emotional/Informational Support Measure

The directions for the support measure asked individuals to state, ‘How often each of the following kinds of support is available to you if you need it’ based on a five-item Likert Scale ranging from ‘none of the time’ to ‘all of the time’. The emotional/informational support scale for this study comprised five items, (Cronbach’s α = 0.95).

Table 2 indicates the frequency with which different types of social support were available to help participants manage stressful issues or situations experienced by their responder partner.

Except for the first statement, ‘someone to confide in’, the remaining questions in this table indicated that the largest percentage of partners claimed to have minimal or a total lack of emotional or information support.

### 3.3. Findings from Thematic Analysis

Participants were asked two open-ended questions: “What more, if anything, would you like the organisation to do for your emergency responder?” and “What more, if anything, would you like the organisation to do for YOU as the partner of an emergency responder?” It became clear that responses directed at supporting the responders also reduced the anxiety that partners carry for the welfare of their emergency responder. The next two sections discuss the overarching themes and the basic themes that emerged from the responses.

#### 3.3.1. Perceived Organisational Support

**Figure 1:**
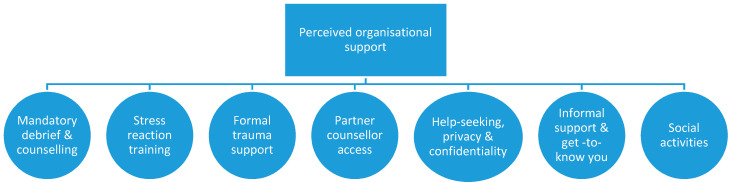
Themes for perceived organisational support.

This theme, as shown in Figure 1, focuses on additional assistance that the partners of the defence/emergency responders want from the organisations to mitigate traumatic stress. The comments from participants address support needed by responders and partners before and after traumatic events.

One of the themes to emerge in this grouping was a belief that *mandatory debrief and counselling* for the responders could help to address what happens after traumatic interactions. Requests for “formal and informal debriefs” included “peer support” programmes, and “compulsory counselling”. There was also an emphasis on “not just if they want to or feel the need”. Another theme emphasised that partners would like *stress reactions training* to help them identify issues for their defence/emergency responders and tools to deal with these reactions once identified. One participant requested “information on ways to support my emergency responder. Do I just listen? How do I know when he needs further help? Where do I go for that help?” Meanwhile, others specifically asked for “more seminars on stress and mental health”.

Another theme referred to *formal trauma support*. This theme addressed getting support after a traumatic event such as providing “follow-up down the track”, a “stand-down period to allow [my responder] to process things and NOT sending them straight to the next job”. Partners also requested “some sort of counselling or professional advice to be available not just for the [responder] but their families” to include couples counselling because “sometimes we are under a lot of relationship stress due to his job”. These requests were part of the theme *partner counsellor access*.

The theme of *privacy and confidentiality* dealt with partners feeling they and their responder would be better supported if organisations addressed the issue of privacy and confidentiality when seeking help for mental health issues and general private family matters. One participant said, “my husband feels it is not safe to seek help with any mental health issues that may arise as this information is not kept confidential”.

Equally important is the theme of *informal support and get-to-know-you events* which encouraged the organisations to add activities or modify current activities to allow partners to connect more effectively with others. For example, requests were made for more family events to share with the children “what Mummy/Daddy do for a job in a positive way”, while some preferred “events for partners of [responders] to bond…that do not include children”. Others suggested “more interaction between wives and girlfriends to create a support network during times the other halves are away” and as a way to meet new people when moving to a new location.

The final theme addressed *social activities* and included specific requests for more sports programmes, “partner sports teams”, and family-friendly sports events. Some partners discussed wanting to have “real-person interactions” and how “social contact would be an advantage in understanding personalities and circumstances of staff”. Some participants who had made connections through the responder’s work discussed the benefits of these types of activities and believe these connections are important. One participant captured the sentiment that several participants articulated: “[organisation] families are the only ones that can truly empathise with what it’s like to be part of a family of a [responder]. It can be incredibly hard sometimes”.

These basic themes expressed by the partners indicated a disconnection between the type of support the organisations are providing and what partners perceive is needed or promised for themselves and their responder for their wellbeing.

#### 3.3.2. Direct Communication and Engagement

**Figure 2:**
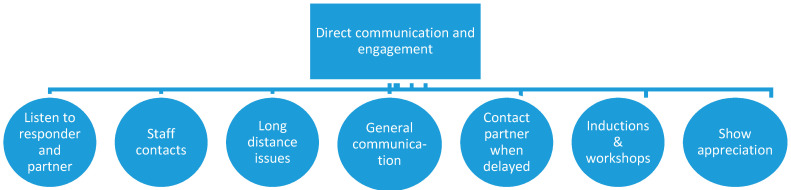
Themes for direct communication and engagement.

The overarching theme, shown in Figure 2, focuses on how the organisations could engage and acknowledge the partners and responders more transparently and directly. These comments describe the experiences and requests of the partners which encourage organisations to engage and communicate with them directly.

The theme of *listening to the responder and partner* was provided by partners who would like to have their own voices heard and suggest the organisations “seek feedback …from the spouses/families of emergency responders”.

A number of responses requested contact details for individuals working in the organisation to find out “who’s who and who to contact if the need arises”. This exemplifies the theme of *staff contacts*.

Different comments made up the theme of *long-distance issues*. Partners who lived in a different town from where the military member or emergency responder works reported related issues including feeling left out, not receiving direct communication, and a lack of understanding about where to get support. One participant living in a different region to where her responder worked, stated, “I didn’t have the support of fellow (organisation) wives. I also don’t have family near, or who understand. The general public doesn’t understand the loneliness and stress”.

Another theme related to *general communication*. Participants want to receive newsletters and emails directly because usually the information does not get forwarded to them by their responder. Other respondents commented on the need to provide Facebook groups and online social networking as another avenue of direct communication.

A request by participants for the organisation to be *in direct contact when their partner’s return home would be significantly delayed* (after a shift or while on deployment) appeared as another theme. One participant said they would appreciate “a phone call if my husband has been called out to a big incident right when his shift has finished”. Another wanted “confirmation that they know I am the person they contact if something was to go wrong in a situation”. Partners would like to be notified when the responder is not involved with routine activities which could be a concern or as one participant said “[that] she has been involved in a serious situation, what state she’s in, approximately when she’ll be home so I can be supportive, be home, go pick her up etc.”.

Another theme involved increased communication with the partners by *providing inductions, workshops* and more information about how to manage life as the partner of a responder. One partner summed this up by saying they wanted “more involvement generally in my life—they should be the ones pointing us in the right direction. At the moment it is up to the individual to hunt around for information and support networks”. Partners wanted the organisations to ensure that “all families get the same information around briefs” and request “briefings for partners, workshops on stress management for families and more active welfare coordinators”. This information could assist them to identify “warning signs and contact numbers for additional help” and “how to manage the stress and day-to-day practicalities of being in a relationship with an emergency responder”.

*Showing appreciation* was the final theme, which exemplified the overarching theme for direct communication and engagement. Partners shared that there was an unmet need to provide “more acknowledgement to families for the massive role they play in supporting the organisation’s biggest asset”. Partners expressed anger “at how underappreciated” they feel and requested “recognition of the role I play in his life that enables him to do his job”.

These themes focus on encouraging the organisation to communicate directly with the partner. Getting direct communication about issues relating to a traumatic event experienced by a responder, personal invites to organisational events, or connections with staff may enhance partners’ ability to make informed life choices.

## 4. Discussion

### 4.1. Secondary Stress and Social Support

This study found that almost 80 percent of the participants have no, little, or mild risk of STS, while around 20 percent appear to be experiencing intrusive, arousal, and avoidance symptoms related to the trauma experienced by their responder. Studies using the STS Scale describe similar results with social workers and welfare workers whose responses met the symptomatic criteria for PTSD [41,42], This prevalence is comparable with the 21 percent found in a study of wives of firefighters [43] and slightly lower than the 28 percent found in a study of police wives [44], although these studies used the Modified Secondary Trauma Questionnaire [45] and not the STS Scale. Other studies of STS in partners of military service members resulted in a wide range from three percent [46] to 10 percent [47], 24 percent [48], and around 30 percent [49]. The variations in the studies could be related to differences in the measures, or due to the specific populations since several of the above studies were with partners of veterans who had long ago left the work environment.

About half of the respondents from this study did not feel they had someone who could provide emotional or informational support to them for their problems or worries because their partner was a military member or emergency responder. Of concern, around 30 percent who felt unsupported stated they had emotional/information support ‘none of the time’. This could be related to a finding which discusses being without support because of issues related to moving location, which put partners into environments far from their usual support networks. Partners also discussed shift work or deployments as barriers to socialising. Family and friends do not always understand how shift work and deployments impact everyday life and the kind of support that would be most useful for the partners. As one participant explained, “it would be nice to talk to other partners [like me] as I only know the odd one that I get introduced to by chance, because my friends don’t understand the shift work or the worry”. Partners see the benefits of connecting with other partners of responders. One partner stated, “I would like to know that partners of other [responders] would be available for discussion if necessary…in case something happened”.

Through the theme of perceived organisational support, partners addressed direct and indirect ways to prevent or mitigate traumatic stress reactions in the responders and partners. Direct strategies address the inclusion of mandatory debrief or counselling after traumatic events; modifications to protocols for how responders are supported after an event; counsellor access for partners of responders who have experienced traumatic events; and educating partners to identify and assist their responder and themselves with stress reactions. Indirect strategies to mitigate traumatic stress reactions include ensuring the confidentiality of responders is respected; decreasing the stigma towards help-seeking; and provision of formal/informal supportive activities to create opportunities for partners to form their own networks with other partners of responders.

Partners are requesting direct communication with the organisation. Communication intended for partners should not rely on indirect methods of communicating (via responders or word of mouth) but should go directly to the partner for all types of events and scenarios. This communication includes induction/workshops/contact information, communication when the responder would have a significant delay in returning home, invitations to any events/activities, and notification of the responder’s involvement in traumatic events. The partners would like the organisations to listen to what they have to say and to express their gratitude to the partners. Direct communication and engagement with the organisation would provide opportunities for partners to make more informed choices in crisis and noncrisis situations.

While the literature is relatively silent on this complex issue, the experiences described in this discussion section have similarly been found in other literature related to partners of emergency responders [5,7,8,9,20,21].

### 4.2. Limitations

There are several important limitations to this study. One limitation is that the cross-sectional design precludes a straightforward assessment of causation. A further limitation of the survey involved recruitment problems. Ultimately, only around six percent of eligible partners took part in the study. The low uptake of the survey in general, specifically by male partners, makes it difficult to determine whether participants represented the entire population. Other possible selection biases include the possibility that participants might disproportionately have been experiencing symptoms of trauma reactions, although it is also possible that those most affected by STS were unable or unwilling to participate. Some participants lacking emotional/informational support might have been motivated to participate in the study. Alternatively, it could be that individuals who have previously benefited from this type of support were more inclined to participate. However, the qualitative analysis has attempted to provide sufficient depth of information for readers to assess the transferability of the study findings.

## 5. Recommendations for Defence and Emergency Responder Organisations

Firstly, organisations need to obtain and maintain updated contact details for partners of their responders. Knowing who the partners are, and enabling organisations to communicate with them, is an essential step towards providing an inclusive environment for partners. The organisation should be very clear about what type of information the partners will receive through this channel and how they can ‘opt out’ or ‘opt back in’ to receive different types of communication. It is incumbent on the organisation to emphasise potential benefits responders and their partners will gain from providing the contact details.

Secondly, organisational support would be enhanced by providing welcome and induction events for the partners of their employees. Such a welcome and induction programme should provide an overview of the organisation, expectations of the responder’s role within the organisation, common challenges facing new partners, where to access information from the organisation and, at the first opportunity, an introduction to other partners within the organisation. Partners ought to be welcomed into new units or sections when the responder changes roles or locations, and provided with an overview of new responsibilities, staff contact details in the new environment as well as an introduction to fellow partners. Organisations must recognise that they may only have a few opportunities to make a good impression on partners, so the invitations should be sincere and inclusive of partners who may feel they do not fit the stereotypical partner mould. These perceptions that organisations care about partners are important for workplace productivity and reducing turnover [50,51]. This study found that these types of engagement with partners minimise psychosocial risks associated with being the partner of a military member or emergency responder.

Thirdly, organisations could share with the partners what programmes are available or what changes are being made within the organisation to address stress reactions, even if, at this time, those programmes offer very little for the partners. All the defence and emergency responder organisations that assisted with this study are acutely aware of the risks regarding primary trauma and their responders. All organisations actively implement or modify programmes and policies to assist their employees, but little attention has been given to having a family component within their induction initiatives. This knowledge of existing programmes or policies enables partners to make informed decisions. It also shares with partners how the organisations are prepared to address traumatic stress issues and can guide partners as to how to help themselves if they are struggling with STS or, at the very least, how to assist their responder if they are struggling with primary trauma reaction. Sharing what the organisations are doing, and the process that individuals go through to get help, can also positively shape the perception the partners have around the competencies of the organisation to address traumatic stress.

Finally, organisations could provide training for partners around managing stress. It should identify the risks of primary stress that responders may experience and how they may affect the partners. It should include strategies known to reduce negative effects of trauma reactions and provide suggestions for managing those reactions should the responder or the partner experience traumatic stress. It ought to counteract the perceptions the responders may hold around stigma towards help-seeking. In addition, the training needs to identify common maladaptive behaviours and offer techniques to build resiliency. This means that the organisations need direct channels that partners can go through to support these types of engagements.

These recommendations suggest that the New Zealand defence and emergency organisations need to acknowledge the risks and accept that partners have an important role to play in achieving the desired outcomes of both the responder and the organisation.

## 6. Conclusions

This study found that some partners of emergency responders are experiencing risks associated with secondary traumatic stress and deficient social support networks. The qualitative aspect of this study supported these findings and suggested that partners of responders need improved access to tools (education, training, and direct communication) from the organisations to act in an informed manner to assist their responder and themselves with issues related to traumatic events. These findings are consistent with many other researchers’ recommendations that defence and emergency responder organisations need to provide different components of support to the partners to mitigate the psychosocial risks associated with being the partner of a military member or emergency responder [5,6,7,20]. This research suggests that following these recommendations will not only reduce the risks for partners but also increase their ability to provide support that benefits their responder and ultimately increase organisational readiness. New Zealand depends on defence and emergency responders being fully ready to maintain the safety, national security, and wellbeing of the public they serve. Protecting the mental health of the partners of military members and emergency responders plays a large part in equipping New Zealand defence and front-line emergency responders for their roles.

## Figures and Tables

**Table 1:** Adapted secondary traumatic stress (STS) scale results.

Possible Secondary Trauma (*n* = 664)	*n*	%
Little or no risk of secondary trauma	377	56.8
Mild risk of secondary trauma	151	22.7
Moderate risk of secondary trauma	57	8.6
High risk of secondary trauma	32	4.8
Severe risk of secondary trauma	47	7.1
Possible post-traumatic stress from secondary exposure (includes moderate, high, and severe)	136	20.5

**Table 2:** Results for emotional/informational support measure.

Emotional/Informational Support Questions	None of the Time %	A Little of the Time %	Some of the Time %	Most of the Time %	All of the Time %
Someone to confide in or talk to about your feelings about your emergency responder’s work (*n* = 652)	22.7	16.4	17.9	22.7	20.2
Someone who gives you good advice about your emergency responder’s work (*n* = 649)	33.3	19.4	16.0	17.3	14.0
Someone to share your most private worries and fears about your emergency responder’s work (*n* = 646)	31.1	19.3	12.5	19.8	17.2
Someone to turn to for suggestions about how to deal with a personal problem brought about because of your emergency responder’s work (*n* = 644)	32.1	20.5	12.4	18.2	16.8
Someone who understands your problems with your emergency responder’s work (*n* = 646)	29.1	19.0	17.3	18.3	16.3
